# Scorpionism in Brazil in the years 2000 to 2012

**DOI:** 10.1186/1678-9199-20-46

**Published:** 2014-10-15

**Authors:** Guilherme Carneiro Reckziegel, Vitor Laerte Pinto

**Affiliations:** Programa de Pós-Graduação em Saúde Coletiva, Faculdade de Ciências da Saúde, Universidade de Brasília, Brasília, Distrito Federal Brasil; Programa Nacional de Controle de Acidentes por Animais Peçonhentos, Ministério da Saúde, Brasília, Distrito Federal Brasil; Laboratório de Epidemiologia e Vigilância em Saúde, Fiocruz, Brasília, Distrito Federal Brasil

**Keywords:** *Tityus*, Scorpion sting, Epidemiological profile, Public health, Brazil

## Abstract

**Background:**

Scorpionism is a serious public health problem in Brazil. Nationwide epidemiological analyses of scorpion stings are scarce. In this context, the present study aims to provide an epidemiological analysis of accidents involving scorpions in Brazil.

**Methods:**

An analytical epidemiological study of the scorpion accidents reported in the Information System for Notifiable Diseases (SINAN) was conducted from 2000 to 2012 in Brazil.

**Results:**

During this period, 482,616 accidents and 728 deaths were reported. The annual average incidence and mortality rates per 100,000 inhabitants were 19.6 and 0.030, respectively, with annual average lethality rate of 0.16%. The highest accident frequency was recorded in male subjects, aged 20–49 years, from September to December and in urban areas, except in the northern region of the country, where accidents were most frequent in June and July, and in rural areas. Males up to age 9 and rural areas were defined as an age group and area for greatest death risk, respectively.

**Conclusions:**

Scorpionism in Brazil is a predominantly urban health problem that mostly affects people at an economically active age. The Northeast and Southeast hold the majority of cases and deaths, as well as the highest annual incidence and mortality averages, but the Central West and North presented the highest average annual lethality rates. The epidemiological changes described in this study highlight the need for intensifying health surveillance actions to prevent scorpion accidents in Brazil.

## Background

Long ago scorpionism was recognized as a public health problem, being labeled by the World Health Organization (WHO) as a neglected health problem, and thus associated with poverty [1–7]. The current incidence of scorpionism is not known, because not all injured people seek medical attention. However, it is estimated that about 1 million accidents occur annually worldwide [8].

Although scorpionism surveillance activities began to be coordinated by the Brazilian Ministry of Health (MS) in 1988, only since 1997 have venomous animal accidents been reported in the Information System for Notifiable Diseases (SINAN), which is currently the official registry system for compulsory notification of health problems and diseases [9].

The incidence of accidents involving scorpions in Brazil has been increasing over the years [10]. Data collected by the National Program for Control of Accidents by Venomous Animals, in the period from 1990 to 1993, revealed about 8,000 accidents per year, with an average annual incidence of three cases per 100 thousand inhabitants. The main records were from the states of Minas Gerais and São Paulo, totaling approximately 50% of notifications, but other states such as Bahia, Rio Grande do Norte, Alagoas and Ceará have been increasing the number of reports. The majority of cases were clinically mild, with a mortality rate of around 0.9%, while most fatal accidents were associated with the specie *Tityus serrulatus*[10]. Between 1988 and 1999, there was an annual average of 6,267 accidents in Brazil [11].

The Brazilian scorpion fauna is quite large, consisting of about 131 species, 23 genera and four families [12]. The scorpions recognized for their medical importance in Brazil belong to the Buthidae family, genus *Tityus*, with four main species recognized for causing clinically serious accidents: *Tityus serrulatus*, *T. stigmurus*, *T. bahiensis* and *T. obscurus* (synonyms: *T. cambridgei* and *T. paraensis*) [1]. They are spread across all geographical regions of Brazil, with *T. serrulatus* (yellow scorpion) being the main cause of serious accidents [13].

Nationwide epidemiological analyses of the scorpion stings are scarce. In this connection, the present study aims to present an epidemiological analysis of accidents involving scorpions in Brazil, in the period from 2000 to 2012.

## Methods

An analytical epidemiological study of the scorpion accidents recorded in SINAN between 2000 and 2012 was conducted in Brazil. The databases were updated through February, 2014, with two versions of SINAN being used: SINAN – Windows version, from 2000 to 2006; and SINAN-Net version, from 2007 to 2012.

In accordance with federal decree n.1271, of June 6^th^, 2014, accidents with venomous animals are classified as requiring a notification to SINAN as a health problem. The Ministry of Health of Brazil (MS) describes each scorpion accident with clinical evidence of scorpion envenomation, with or without identification of the animal causing the accident [14]. The present study considered the total number of registered cases in SINAN as all the accidents having happened in Brazil.

Demographic data were obtained from the 2000 and 2010 censuses, and projections and interpolation for the years 2001–2009, 2011–2012 between censuses, from the Brazilian Institute of Geography and Statistics (IBGE) [15].

The fields of the Notification/Investigation SINAN Form (FNI) for accidents with venomous animals used in the study were: date of accident, date of death, gender, age, race/color, education, injured part of the body, place of the accident. The presentation of the “Education” field differs between the Windows and Net SINAN versions; therefore, it was standardized according to the Windows SINAN FNI in years of schooling. For this purpose, the variables of this field present in the Net SINAN FNI were characterized as: none, corresponding to illiterate; 1–3 years, corresponding to 1^st^ to 4^th^ incomplete elementary school (ES) grade; 4–7 years, corresponding to 4^th^ full year of ES and 5^th^ to 8^th^ grade of incomplete ES; 8 or more years, corresponding to complete ES, incomplete high school, complete high school, incomplete higher education, or complete higher education.

In scorpion stings, local pain is an instant symptom [13, 16–18]. Therefore, to adjust for the inconsistent dates of the accidents, the date of first symptoms was used.

The annual incidence and mortality rates were calculated using the ratio of the absolute number of recorded accidents and the absolute number of recorded deaths, respectively, for the population at risk; and the ratio between the absolute number of recorded accidents and the absolute number of recorded deaths from scorpion envenomation was used to calculate the annual lethality rate. To calculate the average coefficients of incidence, mortality and lethality, we used the arithmetic mean of their annual coefficients.

The software packages TabWin32 3.6b, Epi Info 7.1.3.3 and Microsoft Excel 2010 were used to tabulate the data; Epi Info 7.1.3.3 was used to calculate the relative risk with its respective 95% confidence interval, based on the average lethality rate; TerraView 3.2.0 was used to produce the maps.

Primary data, simple measures of frequency and arithmetic means were employed to produce the description and analysis of the data.

The study was based on secondary data, without access to the patients’ nominal data or anything else that could identify them. The ethical and legal requirements were followed as specified by Resolution 196/96 from the National Health Council.

## Results

There was an increase in the number of reported cases in Brazil during the period of study, from 12,552 in 2000 to 64,027 in 2012, a total of 482,616 cases. The same happened to the number of deaths, which rose from 13 in 2000 to 89 in 2012, with a maximum of 90 in 2009. During the period a total of 728 deaths were recorded (Table 1).

**Table 1:** **Distribution of the absolute number of cases and deaths due to scorpion envenomation recorded in SINAN, and epidemiological indicators by year of occurrence, Brazil, 2000-2012**

Years	Cases	Deaths	Incidence rate ^a^	Mortality rate ^a^	Lethality rate (%)
2000	12,552	13	7.4	0.008	0.10
2001	17,996	38	10.4	0.022	0.21
2002	22,500	49	12.9	0.028	0.22
2003	24,280	49	13.7	0.028	0.20
2004	29,991	40	16.7	0.022	0.13
2005	35,531	45	19.3	0.024	0.13
2006	37,032	24	19.8	0.013	0.06
2007	37,261	61	19.7	0.032	0.16
2008	40,236	85	21.2	0.045	0.21
2009	50,350	90	26.3	0.047	0.18
2010	51,698	67	27.1	0.035	0.13
2011	59,162	78	29.3	0.041	0.14
2012	64,027	89	31.3	0.046	0.15
**Total**	**482,616**	**728**	**–**	**–**	**–**
**Average** ^**b**^	**–**	**–**	**19.6**	**0.030**	**0.16**

^a^Values corresponding to groups of 100,000 inhabitants. ^b^Arithmetic mean.

Data source: SINAN/SVS/MS.

There was an increase of 323% in the incidence rate between 2000 and 2012, with an average for the period of 19.6 accidents per 100,000 inhabitants. The variation in the mortality rate was even higher, with an increase of 475% and an average of 0.030/100,000 inhabitants. The lethality rate in Brazil had the minimum and maximum values of 0.06% in 2006 and 0.22% in 2002, reaching 0.15% in 2012, with an average of 0.16% for the period (Table 1).

Males were more often affected than females both in the number of cases (50.7%) and deaths (56.3%), with a death risk that was 1.26 times higher. Most victims declared themselves to be black (44.7%), the race/color that presented a higher frequency of deaths (50.5%) and a death risk 1.37 times higher than that of whites. Most of the victims had between four and seven years of education (17.8%). The race/color and education fields presented respective incompleteness percentages of 29.8% and 42.9%. About half of the accidents (47%) were in the age group 20–49, and the proportion of deaths was higher victims up to nine years old (53.9%). Individuals of up to four years old had the highest risk of death, which was 10.09 times higher than that of victims aged 15 years or more. Table 2 describes the demographic characteristics and death risk from accidents during the period of study. The injured regions of the body were diverse, with fingers being the most frequent (24%), followed by the feet (20%), hands (16.4%) and toes (9.5%).

**Table 2:** **Demographic characteristics of scorpionism cases registered in SINAN, Brazil, 2000-2012**

Demographic data	Cases		Deaths		Lethality rate (%)	RR (IC 95%)	***p*** value
	n =482,616	%	n =728	%			
**Sex**							
Male	244,593	50.7	410	56.3	0.17	1.26 (1.09-1.45)	*p <0.05*
Female	237,801	49.3	317	43.5	0.13	1	–
Ignored/Omitted	222	0.0	1	0.1	–	–	–
**Race/Color**							
White	117,306	24.3	146	20.1	0.12	1	–
Black^a^	215,938	44.7	368	50.5	0.17	1.37 (1.13-1.66)	*p <0.05*
Yellow	3,988	0.8	9	1.2	0.23	1.81 (0.92-3.55)	*p >0.05*
Indigenous	1,603	0.3	5	0.7	0.31	2.51 (1.03-6.10)	*p >0.05*
Ignored/Omitted	143,781	29.8	200	27.5	0.14	–	–
**Education** ^b^							
None	17,810	3.7	16	2.2	0.09	1.39 (0.78-2.45)	*p >0.05*
1-3 years	53,716	11.1	82	11.3	0.15	2.36 (1.64-3.39)	*p <0.05*
4-7 years	86,127	17.8	83	11.4	0.10	1.49 (1.04-2.14)	*p <0.05*
8 or more	69,524	14.4	45	6.2	0.06	1	–
Not applicable^c^	48,603	10.1	314	43.1	0.65	9.98 (7.30-13.64)	*p <0.05*
Ignored/Omitted	206,836	42.9	188	25.8	0.09	–	–
**Age** ^d^							
< 1-4	33,038	6.8	234	32.1	0.71	10.09 (8.46-12.02)	*p <0.05*
5-9	35,812	7.4	159	21.8	0.44	6.32 (5.19-7.70)	*p <0.05*
10-11	39,123	8.1	72	9.9	0.18	2.62 (2.02-3.40)	*p <0.05*
15 and + ^e^	374,544	77.6	263	36.1	0.07	1	–
*15-19*	42,598	8.8	38	5.2	0.09	–	–
*20-49*	227,028	47.0	153	21.0	0.07	–	–
*50-64*	67,785	14.0	45	6.2	0.07	–	–
*65-79*	31,242	6.5	21	2.9	0.07	–	–
*80 and +*	5,891	1.2	6	0.8	0.10	–	–
Ignored/Omitted	99	0.0	0	0.0	0.00	–	–
**Accident zone**							
Urban Area	297,595	61.7	281	38.6	0.09	1	–
Rural Area	151,579	31.4	419	57.4	0.28	2.92 (2.51-3.40)	*p <0.05*
Ignored/Omitted	33,442	6.9	28	3.8	0.08	–	–

^a^Race/color black = summation of skin colors black and brown [19]. ^b^Education in complete years of study. ^c^Variable is automatically filled when the notified case is less than seven years old. ^d^Age in years. ^e^Age “15 and +” = summation of ages 15–19, 20–49, 50–64, 65–79, 80 and + .

Data Source: SINAN/SVS/MS.

With respect to the accident zone, accidents historically have been more frequently recorded in urban areas (61.6%), ranging from 43.8% in 2000 to 63.2% in 2012. Northern Brazil alone accounted for about two-thirds of accidents in rural areas (62.8%). Most of the recorded deaths in the country were from rural areas (57.4%). The risk of death these areas was 2.92 times higher compared to urban areas (Table 2).

**Figure 1:**
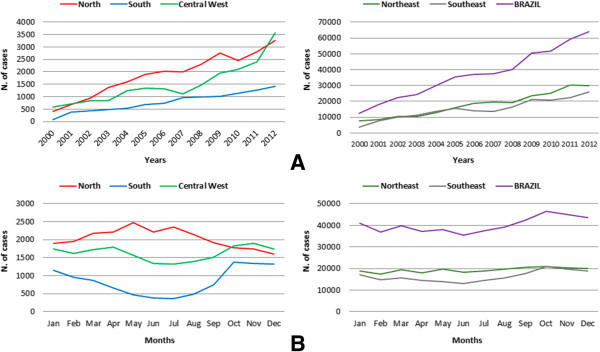
**Temporal distribution of absolute number of cases of accidents with scorpions by region in Brazil by (A) year and (B) month of the accident, Brazil, 2000–2012.**

The accidents were less frequent in the months of June (7.3%) and July (7.7%), peaking in October (9.7%), except in the northern region of the country, where the accidents peaked in May (10.1%) and the lowest rate was registered in December (6.6%) (Figure 1).

Taken together, the Northeast and Southeast presented the highest frequencies of cases (48% and 40.7%) and deaths (43.5% and 44%) (Figure 1A). The states of Minas Gerais (24.2%), Bahia (16.8%), São Paulo (13.3%), Pernambuco (11.1%) and Alagoas (8.7%) had the highest frequency of cases, while deaths were most frequent in Minas Gerais (35.9%), Bahia (30.4%), Pernambuco (6.5%), Pará (5.2%), and São Paulo (3.4%).

**Figure 2:**
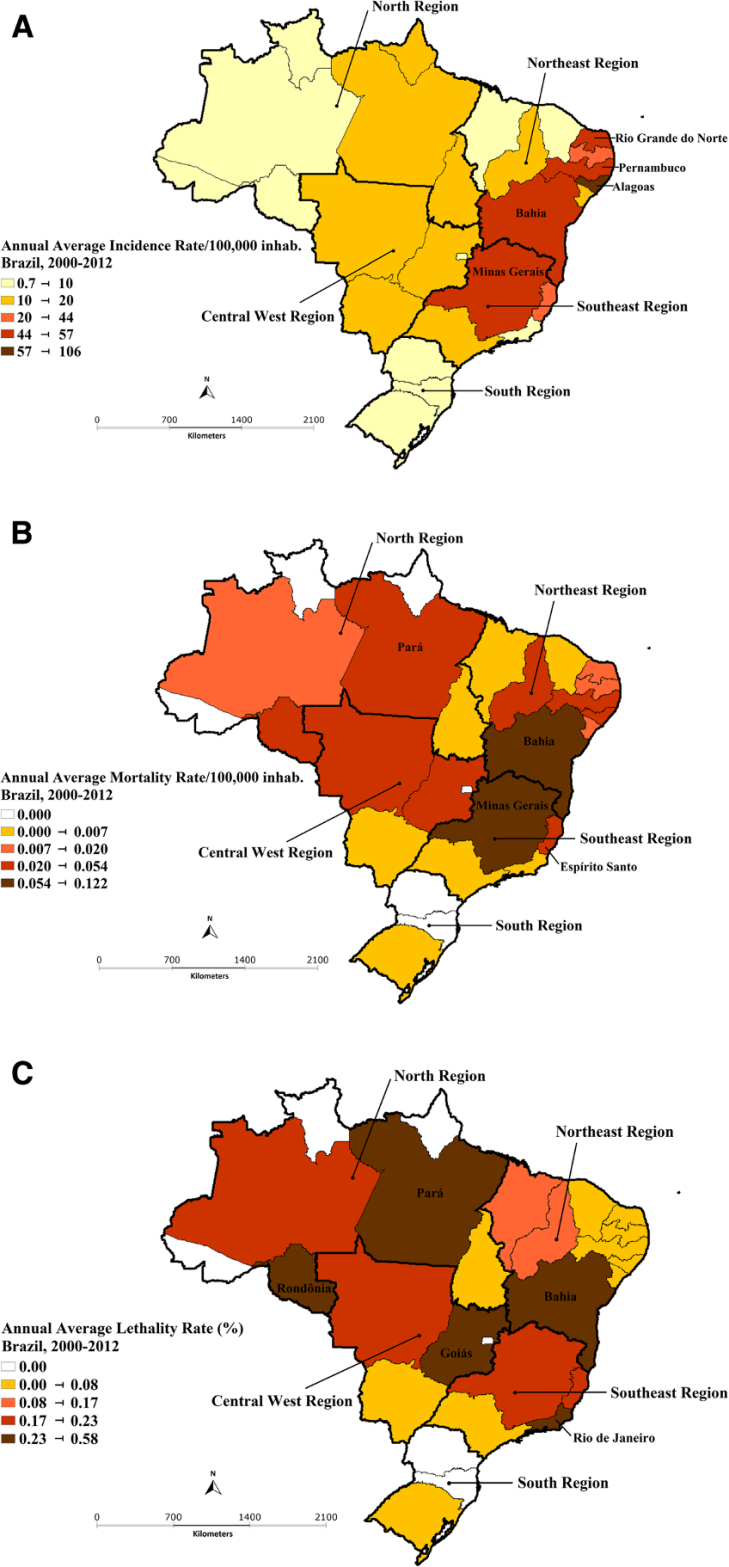
**(A) Annual average incidence, (B) mortality and (C) lethality rates of scorpion envenomation in Brazil, 2000–2012.**

The Northeast and Southeast also presented the highest annual average incidence (Northeast 34.3/100,000 inhabitants; Southeast 19.1; North 12.4; Central West 11.1; South 2.9) and mortality (Northeast 0.047/100,000 inhabitants; Southeast 0.031; North 0.027; Central West 0.021; South 0.000) rates, whereas the North and Central West reported the highest annual average lethality rate (North 0.21%; Central West 0.20%; Southeast 0.16%; Northeast 0.14%; South 0.01%) (Figure 2).

The states with the highest annual average incidence rates were Alagoas (105.9/100,000 inhabitants), Rio Grande do Norte (55.7/100,000 inhabitants), Pernambuco (47.7/100,000 inhabitants), Minas Gerais (46.4/100,000 inhabitants) and Bahia (44.6/100,000 inhabitants) (Figure 2A). The highest annual average mortality rates were Bahia (0.122/100,000 inhabitants), Minas Gerais (0.104/100,000 inhabitants), Espírito Santo (0.053/100,000 inhabitants), Pernambuco (0.041/100,000 inhabitants) and Pará (0.040/100,000 inhabitants) (Figure 2B), Finally, the highest annual average lethality rates were Rondônia (0.58%), Rio de Janeiro (0.36%), Bahia (0.29%), Goiás (0.28%) and Pará (0.24%) (Figure 2C).

## Discussion

The reports of scorpion stings quintupled in the period from 2000 to 2012 in Brazil, especially in the Northeast and Southeast regions, which contributed about 90% of the cases. Males, aged up to nine years old and rural areas were defined as the groups and areas for the greatest death risk, respectively.

The incidence of scorpion stings in different countries varies more widely than that found in the present study [5]. In the southern USA states of Texas and Arizona, the incidence rates are closer to the ones in Brazil, being 22/100,000 inhabitants in both states. Nevertheless, the mortality rate in the southern USA is up to 140 times lower than the one presented in this study, according to Langley [20]. In Latin America, such accidents have been reported in several countries, such as Mexico, Colombia, Venezuela, Argentina and Chile [21–26].

The difference in the accident proportion between males and females was low, probably due to the similar risk exposures in the two genders, based on the characteristics of urban and domestic accident. Studies conducted in smaller groups and/or in different situations showed distinct proportions with regard to this variable, such as Lima *et al*. [27], in Natal (Rio Grande do Norte), with 1,698 accidents, of which 65% affected females; and Pardal *et al*. [28] in Santarém (Pará), with 72 accidents, of which 83.3% were male victims.

Economically active people were more affected, although the mortality rate in this group was lower compared to that of children under 4 years old. This characteristic was also observed in other studies in Brazilian states [29–32]. The emergency treatment of the injured people, mostly children, in reference service centers, is essential to reduce the accidents’ lethality. Access to these centers is more difficult in rural areas, which is an important factor for the higher lethality in these regions.

According to a study performed by Nunes *et al*. [33], household chores, such as handling clothes and wet cloths, expose people to the risk of scorpion accidents, which could explain the higher frequency of accidents in the hands and fingers (40.4%) and feet and toes (29.5%). There are several studies showing such results [34–37].

The growing process of urbanization, poor household conditions and the high ecological plasticity of some medically important scorpion species, such as *T. serrulatus*, referred to as the greatest cause of serious accidents in Brazil, and *T. stigmurus*, have altered the epidemiology of accidents, as evidenced in previous studies [13, 38, 39]. The increasing records of urban accidents over the years corroborate this fact, although most deaths still occur in rural areas. In this context, the most vulnerable socioeconomic populations, people who identify themselves as black and those with lower education levels (between four and seven years of study, equivalent to elementary school), were the most affected. This relation, although described in other studies, needs to be elucidated in Brazil due to possible errors in the completion of the race/color and education variables, which are self-declared [35, 40].

The most populous regions, the Northeast and Southeast, containing about 70% of the population, present respective urbanization degrees of 84.4% and 73.1% [38]. These characteristics, combined with the presence of the perfectly adapted scorpion, are favoring the high incidence rates in these regions. According to Porto and Brazil [39], in tropical countries scorpions are more active in warmer and wetter months of the year, which was also observed in the present study, with an elevated number of accidents at these times, except for the northern region, whose climatic characteristics differ from those in the rest of the country. This feature was discussed by Pardal *et al*. [28] in the municipality of Santarém (Pará), where the highest incidence of scorpion accidents occurred during the months from March to August, confirming the different seasonality in northern locations.

There are several factors that may be linked to deaths from scorpion envenomation including scorpion species causing the accident, lack of access to health care and quality services, and ignorance among the population about the importance of medical care in case of accidents by venomous animals. A study performed by Guerra *et al*. [41] highlights the direct relationship between risk of death and severe cases, and between the time elapsed from the accident to treatment.

Despite the dilution of possible registry errors by virtue of the large number of cases analyzed in the present work, the study was based on a secondary data source supplied by different professionals, allowing different interpretations of the SINAN FNI at the time of its completion. The underreporting is a reality for this health problem, but because of the lack of national comparison systems, there is no other means to measure its magnitude.

The “race/color” and “Education” FNI fields, completed by the victims’ self-declaration, are tenuous because of possible biases in the victims’ definition when declaring their race/color, and in determining the years of study, leading therefore to the possibility of errors in the records, as well as a high degree of incompleteness of these fields.

## Conclusions

The epidemiological changes described in this study highlight the need for intensifying the actions of health surveillance of scorpion accidents in Brazil and also studies that can establish associations between these accidents and environmental risk factors, taking into account regional differences. Additionally, enhancement of the health services is essential to decrease the lethality of these accidents, which constitute a public health problem, especially for the most vulnerable groups.

### Ethics committee approval

All ethical and legal requirements were observed in the present study, as recommended by the Resolution 196/96 from the National Health Council.
